# Siderite precipitation in Paleoarchean oceans during hydrothermal venting

**DOI:** 10.1126/sciadv.ady6851

**Published:** 2025-11-12

**Authors:** Birger Rasmussen, Janet R. Muhling, Nicholas J. Tosca

**Affiliations:** ^1^ School of Earth Sciences, The University of Western Australia, 35 Stirling Highway, Perth, WA 6009, Australia.; ^2^ Department of Earth Sciences, University of Cambridge, Cambridge CB2 3EQ, UK.

## Abstract

Siderite [iron(II) carbonate] is a major constituent of most iron formations. Its origin, which is currently disputed, affects interpretations about the composition of the ocean, atmosphere, and biosphere on early Earth. Direct precipitation from anoxic seawater is unlikely because of its prohibitively slow nucleation rate even in supersaturated solutions. However, recent modeling suggests that siderite may have precipitated at higher temperatures during hydrothermal fluid–seawater mixing in ancient anoxic oceans. Here, we report the presence of iron-rich siderite microparticles (<1.0 micrometers) in 3.49- to 3.25-Ga exhalative iron cherts from the Pilbara Craton, Australia, which we interpret to have formed during venting of iron(II)-rich hydrothermal fluids into anoxic water columns. Geochemical modeling suggests that coexisting magnesium-rich siderite formed in solutions dominated by seawater. The lack of hydrothermal iron siderite and predominance of magnesium siderite in younger analogs indicate that the bulk of siderite in iron formations is diagenetic and formed from marine pore fluids.

## Teaser

Seafloor hydrothermal venting of iron on early Earth may have triggered seawater precipitation of iron carbonate crystals.

## INTRODUCTION

Siderite (FeCO_3_) is a major phase in iron formations, and its origin, whether a direct precipitate from seawater or a diagenetic phase, has implications for the chemistry of the ancient oceans and atmosphere. For instance, if the siderite in >1.8-Ga iron formations precipitated directly from CO_2_-rich seawater, then concentrations of atmospheric CO_2_ may have been up to 100 times higher than today (*1*). By contrast, if siderite precipitated from anoxic bottom waters in stratified oceans [e.g., (*2*–*7*)], then it would not provide a proxy for atmospheric CO_2_ but could inform estimates of dissolved inorganic carbon (DIC) in the ancient ocean. Recent experiments and thermodynamic modeling suggest that siderite is unlikely to have originated via direct precipitation from ancient seawater (*8*, *9*) and that its growth is exceedingly slow even at very high partial pressure of CO_2_ levels (*8*, *10*). These results cast doubt on the precipitation of siderite or amorphous Fe carbonate at low temperature in early anoxic oceans.

Carbon isotopes may provide clues to the genesis of siderite in Archean-Paleoproterozoic banded iron formations (BIFs) but they are also subject to multiple interpretations. Iron carbonates in BIFs have negative δ^13^C-isotopic compositions [−2 to −14 per mil (‰)], which are widely taken to indicate an important role for dissimilatory iron reduction (DIR) in their formation (*11*, *12*). Specifically, siderite is thought to grow in sediment porewaters during the partial oxidation of δ^13^C-depleted organic matter and microbial reduction of primary precipitates comprising Fe(III) oxides/hydroxides. However, it has also been suggested that the ^13^C-isotopic composition may reflect variable contributions from ^13^C-depleted (δ^13^C; –6.5‰) mantle sources released at hydrothermal vents (*1*, *3*, *5*, *13*) or reflect kinetic isotope effects during abiotic FeCO_3_ precipitation from supersaturated solutions (*14*).

**Fig. 1.**
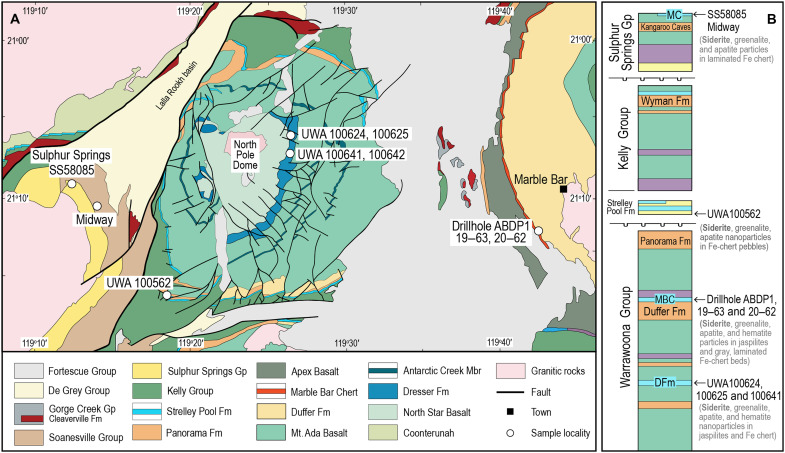
Map and stratigraphic column of the area around the North Pole Dome in the East Pilbara Terrane, Western Australia. (**A**) Simplified geological map of the North Pole Dome area showing sample localities. Fm, Formation; Gp, Group; Mbr, Member. (**B**) Stratigraphic column showing chert horizons sampled in this study. DFm, Dresser Formation; MBC, Marble Bar Chert; MC, Marker Chert unit.

An alternative model, based on recent geochemical modeling simulating hydrothermal alteration of early Precambrian seafloor basalts, posits that siderite may have precipitated at higher temperatures (~100°C) upon mixing of anoxic, sulfate-free seawater with effluent from hydrothermal vents (*15*). To investigate whether hydrothermal siderite formed around seafloor vents in the early oceans, we studied well-preserved 3.49- to 3.25-Ga exhalative cherts from the East Pilbara Terrane, Pilbara Craton, Australia (Fig. 1). We report the presence of two distinct siderite populations, namely, (i) minute (typically <1.0 μm), Fe-rich crystals distributed among nanoparticulate greenalite and apatite and (ii) larger (5 to 20 μm) Mg-rich rhombs and aggregates. Leveraging available thermodynamic constraints on the (Fe,Mg)CO_3_ solid solution as a function of temperature, we argue that the Fe-rich siderite microcrystals precipitated from fluids dominated by hydrothermal effluent, whereas the Mg-rich rhombs formed from pore fluids dominated by seawater. Notably, major BIFs and Fe-rich cherts from the Hamersley and Transvaal basins (2.6 to 2.4 Ga) lack Fe-rich siderite microcrystals but contain abundant Mg-rich siderite aggregates that likely formed in sediment porewaters.

## RESULTS

### Samples and geological setting

Samples of ferruginous chert were collected from multiple localities and stratigraphic horizons in the East Pilbara Terrane, Pilbara Craton, Western Australia (Fig. 1). Sampled intervals include the ~3.49-Ga Dresser Formation, the ~3.46-Ga Marble Bar Chert Member (Duffer Formation), the 3.43- to 3.35-Ga Strelley Pool Formation, and the ~3.25-Ga Marker Chert unit of the Kangaroo Caves Formation (Fig. 1). Polished thin sections prepared from the samples were examined by petrographic microscope, scanning electron microscope (SEM) with energy-dispersive x-ray spectrometer (EDS), and electron probe microanalyzer (EPMA) with five wavelength-dispersive x-ray spectrometers (WDS). Foils, 8 μm by 8.5 µm, were cut from the polished thin sections and thinned to ~100 nm by focused ion beam (FIB) milling. The foils were characterized by transmission electron microscope (TEM) and scanning TEM (STEM) imaging and STEM-EDS.

#### Dresser Formation, Warrawoona Group

The Dresser Formation overlies the 3.53- to 3.49-Ga North Star Basalt and is disconformably overlain by the ~3.47-Ga Mount Ada Basalt. The formation comprises a chert-barite unit up to 50-m thick that is underlain and overlain by basaltic flows and subvolcanic sills (*16*). The chert-barite unit contains silicified volcaniclastic mudstones and sandstones, beds of banded barite and laminated chert (typically jasper) interpreted to include primary chemical precipitates. Barite bands within the chert are linked to barite in vertical hydrothermal chert veins, which originate in altered basalts underlying the Dresser Formation. The upper and lower contacts between the chert-barite unit and basalts are nonerosional, indicating that there was no major time break between extrusion and deposition.

The depositional environment has been interpreted to be shallow marine, intertidal to supratidal, with possible evaporative lakes and terrestrial hot springs (*17*, *18*). Others have argued for a predominantly submarine hydrothermal environment, possibly within a volcanic caldera, in moderate to shallow water depths (*19*). The chert-barite unit preserves laminated and domal structures resembling stromatolites and simple carbonaceous structures interpreted by some to be possible microfossils (*20*–*22*). The presence of bands of fibrous quartz, interpreted to be fossilized pallisade fabrics (*18*), would imply the presence of filamentous microbes.

Samples of red, pink, and gray laminated chert were collected from the chert-barite unit along the eastern flank of the North Pole Dome (Fig. 1). In this area, the Dresser Formation comprises thin sequences of shallowly dipping, silicified volcano-sedimentary rocks that have undergone only low strain and low-grade metamorphism (<350°C). It is generally thought that the banded Fe cherts are silicified chemical muds deposited from submarine hydrothermal vent plumes (*17*, *18*, *23*–*25*). Details about sample localities can be found in (*17*) and (*25*).

#### Marble Bar Chert Member, Warrawoona Group

The ~3.46-Ga Marble Bar Chert Member is a 50- to 150-m-thick succession of red, white, and gray chert in the uppermost Duffer Formation, which is a 100- to 8000-m-thick succession of felsic volcanic and volcaniclastic rocks. The Marble Bar Chert Member comprises a largely fining upward sequence of silicified conglomerate, breccia, sandstone, siltstone, shale, carbonate rocks, fine-grained volcaniclastic rocks, and bedded hydrothermal units, including jaspilitic chert. It is crosscut by vertical veins of dark gray and black chert interpreted to represent the former fracture networks of hydrothermal fluids (*16*).

Samples were collected from the Archean Biosphere Drilling Project drillhole #1 (ABDP1) (Fig. 1), which intersected 100 m of the Marble Bar Chert Member (*26*, *27*). The samples include jaspilitic chert and gray laminated Fe-rich chert, interpreted to have been deposited from submarine hydrothermal vents (*26*). Details of the samples can be found in (*27*–*29*).

#### Strelley Pool Formation

The 3.43- to 3.35-Ga Strelley Pool Formation is a ~100-m-thick unit of variably silicified conglomerate, sandstone, gray- and white-banded shale, carbonate, felsic tuffaceous and volcaniclastic conglomerate, and sandstone. It is locally intruded by veins of hydrothermal, black-gray chert that terminate in the unit. The formation preserves a broad range of laminated and domal structures interpreted to represent some of Earth’s oldest fossilized stromatolites (*30*, *31*).

The sample (UWA100562) was collected from the southwestern portion of the North Pole Dome area (Fig. 1), where the Strelley Pool Formation is typically ~30-m thick, overlies felsic volcanic and volcaniclastic rocks of the ~3.43-Ga Panorama Formation and Mt. Ada Basalt (Warrawoona Group), and is overlain by the 3.35- to 3.31-Ga Euro Basalt (Kelly Group) (*16*, *31*, *32*). The sample is from a basal chert-boulder conglomerate and sandstone (*17*), which is interpreted to be a transgressive, shallow marine deposit (*30*, *31*). Among clasts of felsic volcaniclastic rocks and hydrothermal black and gray chert are fragments of dusty Fe-rich chert. Sample details and descriptions can be found in (*33*).

#### Kangaroo Caves Formation, Sulphur Springs Group

The ~3.25-Ga Kangaroo Caves Formation is a ~1.5-km-thick succession of basalt, andesite, dacite, and rhyolite. It is the uppermost unit of the Sulphur Springs Group and overlies either ~2.4 km of komatiite and komatiitic basalt (Kunagunarrina Formation) or clastic sedimentary rocks of the Leilira Formation (*16*). The top of the Kangaroo Caves Formation contains a laterally continuous chert (informally the “Marker Chert” unit). The chert unit is locally up to 100-m thick and comprises silicified fine-grained volcaniclastic rocks, sandstone, ferruginous chert, and veins of black hydrothermal chert. Volcanogenic massive sulfide (VMS) mineralization occurs in the top of the Kangaroo Caves Formation and below the Marker Chert. Samples of ferruginous chert were collected from diamond drill core of the ~3.25-Ga Marker Chert unit from the main Sulphur Springs VMS deposit and the Midway prospect (Fig. 1).

### Siderite in Paleoarchean Fe cherts

#### Chert

**Fig. 2.**
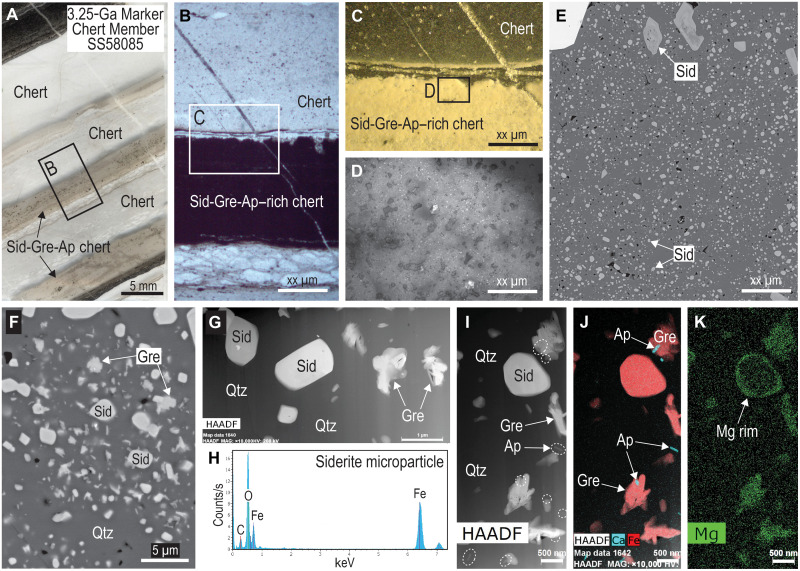
Optical and electron microscope images of siderite in ferruginous chert from the Marker Chert unit, Kangaroo Caves Formation, Sulphur Springs VMS deposit. (**A**) Polished thin section of ferruginous chert with bands of very fine-grained siderite (Sid), greenalite (Gre), and apatite (Ap). (**B**) Plane-polarized light (PPL) image of particle-free chert (white) and particle-rich chert (dark). (**C**) Image of area in (B) showing that the particle-rich band is white in incident light due to internal reflections in carbonate inclusions. (**D**) Reflected light image showing that the bright band (C) contains numerous minute (light gray-white) particles in chert (gray). (**E** and **F**) Backscattered electron images of Fe-rich chert showing abundant minute particles, including Sid and Gre dispersed in quartz (Qtz). (**G**) High-angle annular dark-field (HAADF) STEM image of Sid euhedra and Gre aggregates in FIB foil from Fe-chert band. (**H**) STEM-EDS spectrum of Sid microcrystal in FIB foil. (**I**) HAADF STEM image of Sid euhedra in Qtz cement with abundant Gre and Ap (white hatched outlines) particles in FIB foil. (**J** and **K**) STEM-EDS element maps for Fe and Ca (J) and Mg (K). MAG, magnification; HV, high voltage.

**Fig. 3.**
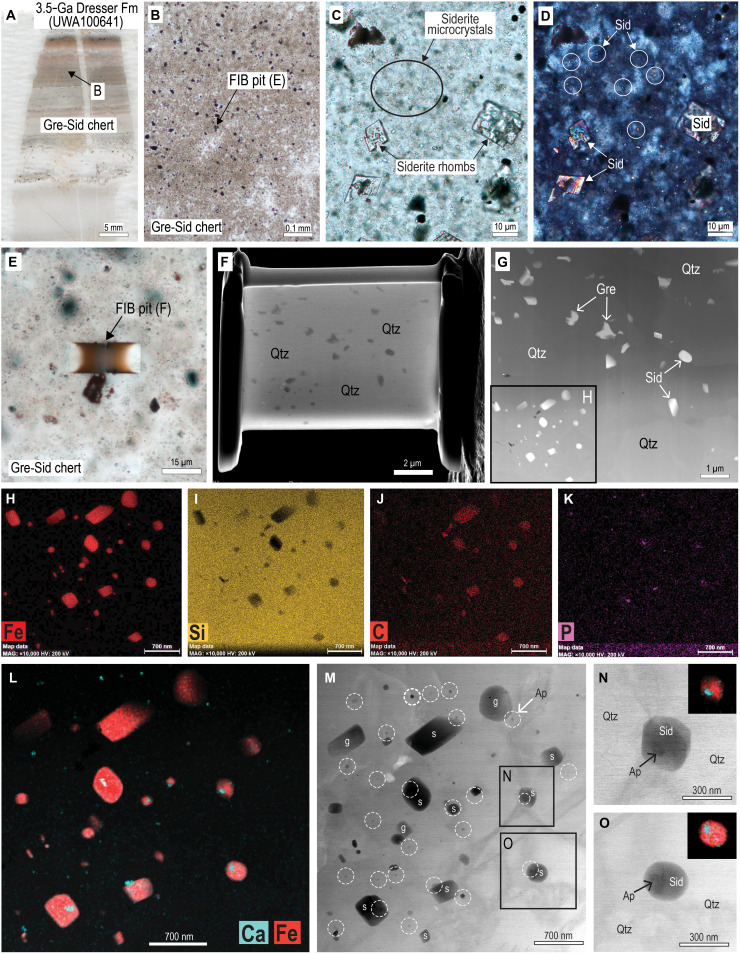
Optical and electron microscope images of siderite (Sid) in ferruginous chert from the Dresser Formation. (**A**) Polished thin section of laminated ferruginous chert with beds of very fine-grained Sid, greenalite (Gre), and apatite (Ap). (**B**) PPL image showing location of FIB pit in siderite- and greenalite- rich chert (Sid-Gre-chert). (**C**) PPL image of particle-rich chert with numerous minute inclusions between larger Sid rhombs. (**D**) Cross-polarized light image of (C) showing highly birefringent Sid microcrystals and rhombs in chert cement. (**E**) PPL image showing location of FIB pit in particle-rich chert. (**F**) Secondary electron image of FIB foil removed from pit in (E). (**G**) HAADF STEM images of Sid and Gre dispersed in quartz (Qtz) cement in FIB foil [see (F)]. (**H** to **K**) STEM-EDS element maps for Fe (H), Si (I), C (J), and P (K). Minute bright specks in P map are interpreted to be Ap. (**L**) STEM-EDS element map (Fe: red; Ca: cyan) showing close spatial association between Sid and Ap crystals. (**M** to **O**) Bright-field TEM images of siderite (“s”), greenalite (“g”), and apatite nanoparticles in chert cement. Apatite nanoparticles are circled in panel (M).

Microcrystalline quartz comprises up to 90 to 95 vol % of the Fe-chert samples (Figs. 2 and 3). The cherts, however, are rarely composed entirely of quartz and commonly contain pyrite, siderite, and ankerite-ferroan dolomite, as well as very fine-grained particles of hematite and greenalite. Surface samples display evidence for weathering, including dissolution and/or oxidation of Fe-rich phases [see (*25*, *33*)].

#### Siderite

Laminated ferruginous cherts from the Dresser Formation and Marble Bar Chert Member (Warrawoona Group), Marker Chert unit (Sulphur Springs Group), and chert-pebble conglomerates in the Strelley Pool Formation (Fig. 1) contain abundant, minute (typically <1 μm), equant crystals (Figs. 2 and 3). The microcrystals have high relief and birefringence (Fig. 3D), consistent with a carbonate mineral. STEM-EDS shows that they are mainly composed of Fe, C, and O (Fig. 2H) characteristic of siderite (FeCO_3_). The chert beds also contain larger (5 to 20 μm) crystals and aggregates of siderite (Figs. 2E and 3C) and large (<1 mm), zoned euhedral “porphyroblasts” of ankerite-ferroan dolomite.

**Fig. 4.**
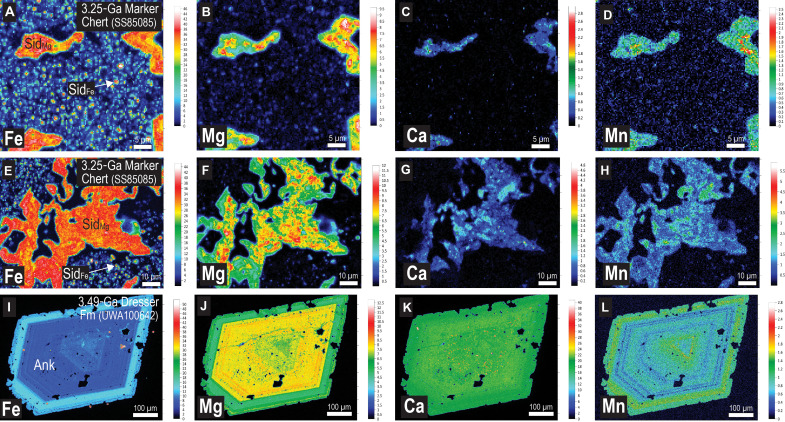
Quantitative electron microprobe (WDS) element maps (in weight %) for Fe carbonates in Paleoarchean Fe chert. (**A** to **D**) Element maps for siderite microcrystals (Sid_Fe_) and rhombs (Sid_Mg_) in Fe chert; Fe (A), Mg (B), Ca (C), and Mn (D). (**E** to **H**) Element maps for siderite aggregates (Sid_Mg_) in Fe chert; Fe (E), Mg (F), Ca (G), and Mn (H). (**I** to **L**) Element maps for ankerite-ferroan dolomite (Ank) crystal; Fe (I), Mg (J), Ca (K), and Mn (L). (A) to (H): Marker Chert unit, Kangaroo Caves Formation; (I) to (L): Dresser Formation.

**Fig. 5.**
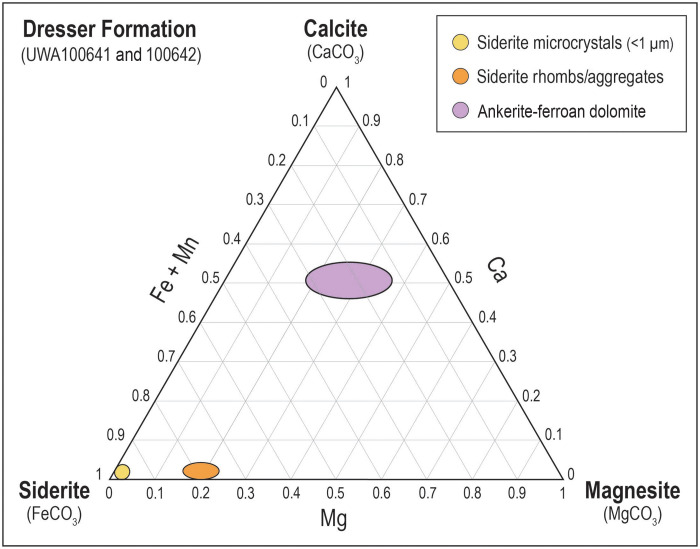
Ca-Mg-Fe + Mn ternary diagram showing the chemical composition of Paleoarchean carbonates. Data plotted (atomic proportions in the cation site) for siderite microcrystals, siderite rhombs/aggregates, and ankerite-ferroan dolomite crystals in laminated Fe chert from the Dresser Formation.

The siderite microparticles in all samples are chemically distinct from the larger siderite rhombs (Figs. 4 and 5). The cations in the submicron-sized siderite are almost entirely Fe, with little or no Mg, Ca, and Mn, whereas in the Dresser Formation, larger siderite rhombs and aggregates contain 37 to 39 wt % Fe and up to 5 wt % Mg (~22 mol % MgCO_3_), 0.4 wt % Ca, and 2 wt % Mn (Fig. 4). Quantitative WDS element maps of siderite aggregates from the Marker Chert unit indicate up to 10 wt % Mg (~43 mol % MgCO_3_) and show complex compositional zoning (Fig. 4, E to H), suggesting multiple episodes of precipitation and partial dissolution. In contrast, larger euhedral ankerite-ferroan dolomite crystals display simple concentric zoning (Fig. 4, I to L).

**Fig. 6.**
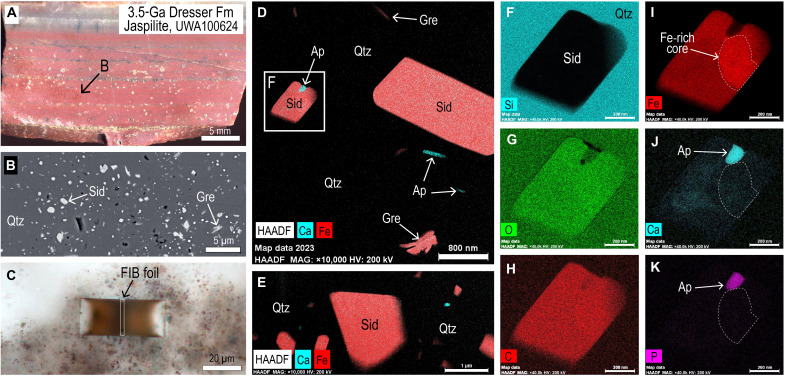
Optical and electron microscope images of siderite (Sid) and apatite (Ap) in jaspilite from the Dresser Formation. (**A**) Polished hand specimen of jaspilite. (**B**) Backscattered electron image of dusty chert containing minute particles of siderite (Sid), greenalite (Gre), and hematite (white). (**C**) PPL image showing location of FIB pit in dusty chert. (**D** and **E**) STEM-EDS element maps (Fe: red; Ca: cyan), showing distribution of Sid, Gre, and Ap in quartz (Qtz) cement. (**F** to **K**) STEM-EDS element maps for siderite-apatite (Sid-Ap) composite grain; Si (F), O (G), C (H), Fe (I), Ca (J), and P (K).

Siderite microcrystals are dispersed in chert cement and rarely share grain boundaries with other siderite grains (Figs. 2, 3, and 6). Most siderite microparticles are subhedral to euhedral (Fig. 6), although some smaller siderite grains (<0.5 μm) in the Dresser Formation are semispherical (Fig. 3, L to O). In all Fe-chert and jaspilite samples examined by TEM, siderite microparticles co-occur with greenalite and apatite nanoparticles (Figs. 2, 3, and 6), with additional very fine-grained hematite euhedra in jaspilites. STEM-EDS element mapping shows that some of the larger siderite microcrystals have a thin rim with elevated Mg content (Fig. 2K). This is most notable in samples of the Marker Chert unit (Sulphur Springs Group).

#### Greenalite

Siderite microcrystals occur with aggregates of randomly oriented greenalite plates in TEM foils of Fe chert (Figs. 2, 3, and 6). Greenalite also occurs as smaller, isolated nanoparticles (<0.5 μm long) within the chert cement. STEM-EDS spectra show that greenalite particles are mainly composed of Fe, Si, and O, with trace amounts of Mg and Al. The platy crystals have a (001) *d*-spacing ~0.73 nm and a 2.2-nm modulated superlattice characteristic of greenalite (*34*). In Fe-chert pebbles from the Strelley Pool Formation, greenalite particles define polygonal structures separated by particle-free chert. These textures are found in younger Fe cherts and BIFs, where the particle-free chert is interpreted to form by filling cavities that developed during dehydration and recrystallization of hydrous silica cement (*33*, *35*).

#### Hematite

Very fine-grained hematite crystals are present in all jaspilites (i.e., Dresser Formation and Marble Bar Chert Member) but are rare or absent in gray-green laminated chert and Fe-chert pebbles. Light microscopy shows that jaspilites contain minute particles (typically <2 μm) of hematite dispersed in microcrystalline quartz. The hematite is commonly concentrated into polygonal structures separated by hematite-free quartz similar to those described above and defined by greenalite. They are interpreted to represent silica shrinkage structures (*36*–*39*). In most Fe-chert and jaspilite samples, hematite is less abundant than greenalite nanoparticles and siderite microcrystals.

#### Apatite

Tiny, randomly oriented, prismatic crystals, between 10 to 400 nm long, are scattered among larger siderite, greenalite, and hematite crystals in the Fe-chert samples (Figs. 2, 3, and 6). The crystals are composed of Ca, P, and O, with minor F, indicating that the mineral is probably fluorapatite. The apatite nanoparticles are mostly enclosed in quartz cement but may also abut greenalite and siderite particles and, in some cases, are enclosed in greenalite (Fig. 2, I and J) or, more commonly, siderite microcrystals (Fig. 6).

### Siderite in Neoarchean–early Paleoproterozoic BIF

**Fig. 7.**
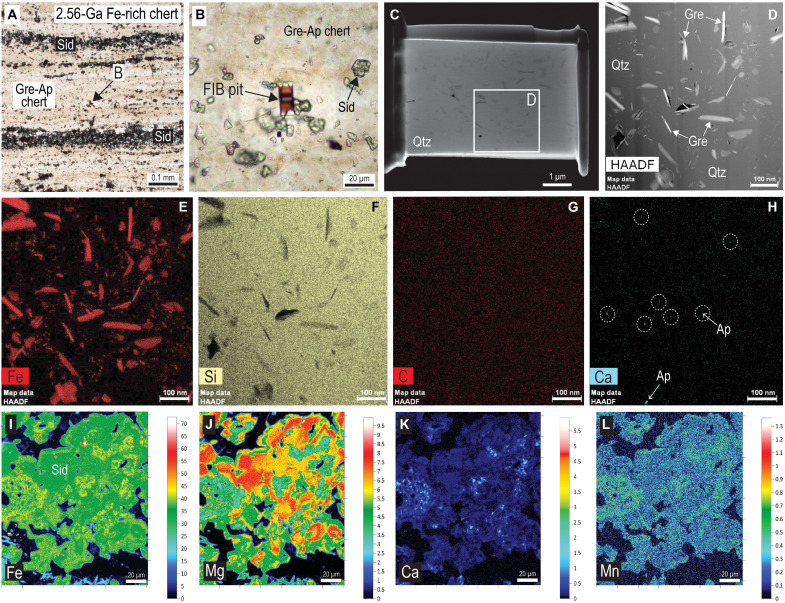
Optical and electron microscope images and element maps of greenalite (Gre) and siderite (Sid) in ferruginous chert. (**A**) Polished thin section of ferruginous chert comprising siderite-rich (Sid) laminae and greenalite- and apatite-bearing (Gre-Ap) chert bands. (**B**) PPL image showing location of FIB pit in Gre-rich chert. (**C**) Secondary electron image of FIB foil removed from pit in (B). (**D**) HAADF STEM image of randomly oriented Gre plates dispersed in quartz (Qtz) cement [see (C)]. (**E** to **H**) STEM-EDS element maps for Fe (E), Si (F), C (G), and Ca (H). Bright specks in Ca map (circled and arrow) correspond with minute particles of Ap. (**I** to **L**) Quantitative electron microprobe (WDS) element maps (in weight %) of Sid aggregates concentrated along laminae; Fe (I), Mg (J), Ca (K), and Mn (L). Sample is from the ~2.56-Ga Wittenoom Formation, Hamersley Group, collected from drillhole ABDP9, with drill depth of 190.98 to 191.05 m.

Submicron-sized siderite crystals are rare or absent from FIB foils of ferruginous cherts and BIFs from Western Australia and South Africa deposited between 2.60 to 2.40 Ga (*29*). Instead, greenalite and apatite are the only abundant nanoparticulate phases in FIB foils of well-preserved laminated chert beds (Fig. 7), with hematite present in jaspilitic bands. However, coarser-grained Mg-rich siderite is abundant and occurs as larger rhombs (10 to 30 μm) and aggregates of crystals. In contrast to greenalite and apatite nanoparticles, which are randomly distributed in chert laminae, siderite rhombs and intergrowths are typically concentrated along lamina surfaces (Fig. 7). The siderite aggregates contain up to 8 wt % Mg (~38 mol % MgCO_3_) and 43 wt % Fe, with minor Ca and Mn (Fig. 7), similar to coarser-grained siderite in Paleoarchean cherts (Fig. 4). The element maps of siderite aggregates in some BIF samples (Fig. 7) reveal complex zoning, hinting at two or more episodes of siderite precipitation.

## DISCUSSION

### Origin of Fe-rich siderite microparticles in Paleoarchean chert

Siderite in Paleoarchean cherts has previously been linked to (i) direct precipitation from deep anoxic seawater in a stratified ocean (*6*, *40*), (ii) precipitation in a submarine volcanic caldera during enhanced magmatic degassing (*7*, *41*), and (iii) early diagenetic growth in porewater enriched in DIC (*26*). However, limited microtextural or compositional information about the siderite in these occurrences hampers interpretation of its origin. Micron-sized siderite crystals were first documented in a TEM study of jaspilites of the Marble Bar Chert Member in drillhole ABDP1 (*26*). It was argued that the fine-grained hematite was a primary precipitate that formed at 60°C near seafloor vents following mixing between hydrothermally emitted Fe^2+^ and oxygenated seawater. The presence of dissolved oxygen was interpreted to indicate the existence of oxygenic photosynthesizers at 3.46 Ga. Fine-grained siderite and magnetite in the jaspilites were interpreted to have formed after hematite following the consumption of dissolved oxygen by organic matter and/or dissolved Fe^2+^ in sediment porewaters (*26*). However, the textural arguments for replacement of primary hematite by siderite and magnetite are equivocal. Given the evidence that at least some of the hematite formed after deposition [cf. (*27*, *28*)], it is plausible that none of the hematite is primary but the product of secondary oxidative processes such as those that formed hematite in the Apex Basalt deeper in drillhole ABDP1 (*42*).

By contrast, several lines of evidence suggest that the fine-grained, Fe-rich siderite microparticles identified in Fe-bearing Paleoarchean cherts (Fig. 1) precipitated in mixtures of hydrothermal vent effluent and seawater. First, the Fe-chert beds were deposited in environments associated with vigorous seafloor hydrothermal alteration, sandwiched between thick sequences of mafic and, less commonly, felsic volcanic rocks. The rare earth element and yttrium patterns of the Paleoarchean Fe-bearing cherts have pronounced positive Eu anomalies, typical of chemical sediments interpreted to have been deposited from hydrothermally modified seawater (*7*).

In addition, the minute size of the siderite microcrystals (typically <1 μm) overlaps with the size distribution of Fe-rich particles in modern hydrothermal plumes [0.45 to 2.0 μm; (*43*)]. The siderite microcrystals are dispersed in chert and, unlike coarser-grained siderite rhombs and aggregates, rarely share grain boundaries but typically “float” in quartz cement. The texture and distribution of the siderite microcrystals in the chert cements are consistent with the microfabrics of suspended mud deposits with initial porosities > 70%, which were silicified on or immediately below the seafloor before sediment compaction.

### Geochemical constraints

Qualitatively, the Fe-rich composition of the siderite microcrystals points to a hydrothermal origin. Modern hydrothermal vent fluids contain up to 24 mmol/kg of Fe^2+^(aq) but little or no Mg^2+^ (*44*, *45*). In contrast, modern seawater contains about 53 mmol/kg of Mg^2+^. The consistently high Fe content of the siderite microcrystals (Fig. 5) supports derivation from Fe-rich vent fluids, with minimal mixing with Mg^2+^-rich seawater.

**Fig. 8.**
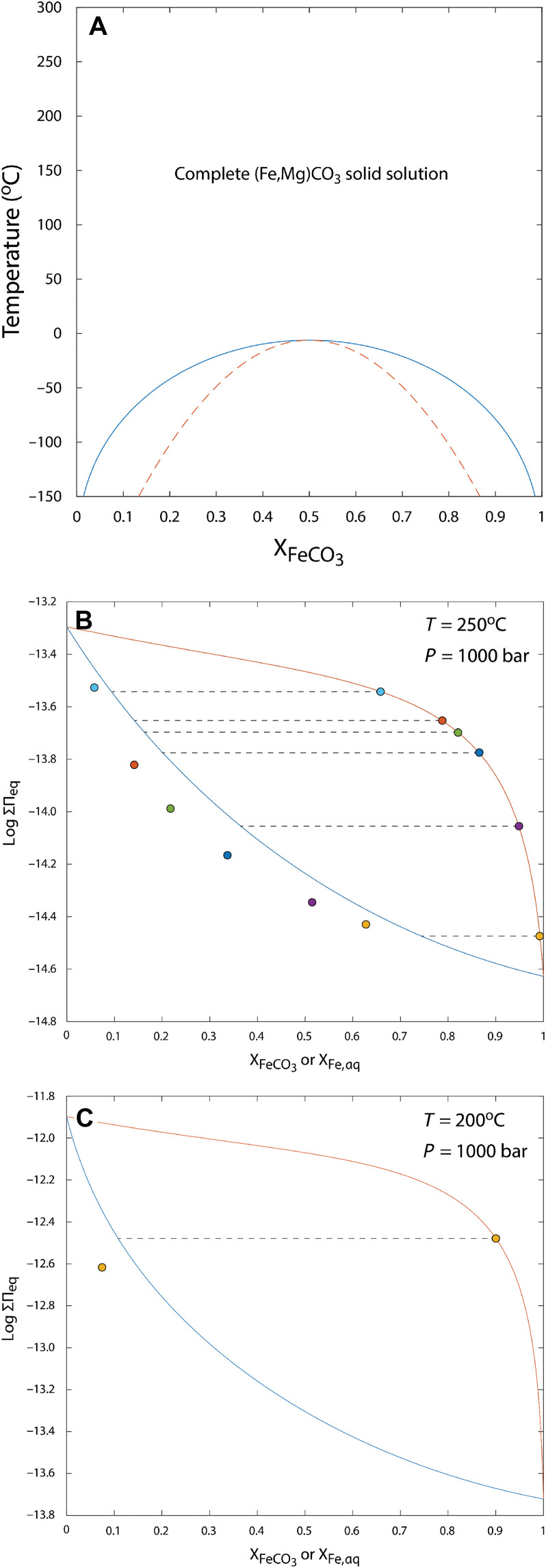
Thermodynamic stability of (Fe,Mg)CO_3_ as a function of temperature and aqueous fluid composition. (**A**) Phase diagram for the (Fe,Mg)CO_3_ system as a function of mole fraction of FeCO_3_ (X_FeCO3_) [after (*46*)]. The solid curve corresponds to the solvus curve, which demarcates the miscibility gap, and the dashed curve corresponds to the spinodal curve, below which spinodal decomposition becomes possible. (**B** and **C**) Solid solution–aqueous solution equilibria for (Fe,Mg)CO_3_ solids at 250°C (B) and 200°C (C) and 1000 bar. The “solidus” curve, shown in red, indicates the thermodynamic solubility of (Fe,Mg)CO_3_ solids as a function of mole fraction of FeCO_3_ (X_FeCO3_). The “solutus” curve, shown in blue, indicates coexisting aqueous solution composition (in terms of the activity fraction of Fe relative to Mg, or X_Feaq_) in equilibrium with a solid of a given composition. Data points for solid and aqueous solution compositions are from (*47*), with X_Feaq_ calculated using procedures described in the Supplementary Materials. Dashed lines indicate tie lines between coexisting solid and aqueous solution compositions, with data points from the same experiment denoted in the same colors.

Available thermodynamic data place quantitative constraints on the stability of compositions in the (Fe,Mg)CO_3_ solid solution in aqueous fluids. First, calorimetric studies of the (Fe,Mg)CO_3_ system have shown that complete solid solution is thermodynamically favored at temperatures above –6°C (Fig. 8A) (*46*). These constraints, along with the measured solubilities of endmember FeCO_3_ (siderite) and MgCO_3_ (magnesite) at high temperature, permit the determination of equilibrium conditions between aqueous solutions and (Fe,Mg)CO_3_ solids across a range of temperature (see the Supplementary Materials). Together, these data show that across a wide range of temperature, (Fe,Mg)CO_3_ solids with appreciable Mg content (i.e., 10 mol % and higher) become thermodynamically stable only in fluids where the activity fraction of Mg far outweighs that of Fe. Although data are somewhat limited, previous hydrothermal experiments between 200° and 500°C and 1000 bar in the Mg-Fe-CO_3_-Cl-H_2_O system (*47*) show reasonable agreement with these thermodynamic predictions and together indicate that (Fe,Mg)CO_3_ compositions approach thermodynamic predictions at least under hydrothermal conditions (Fig. 8, B and C) (Supplementary Materials).

To place these constraints in a process-oriented framework, we executed geochemical reaction path models originally developed by (*15*) representing the mixing between hydrothermal vent fluids and seawater across a range of parameters. These models are designed to represent three major components of subseafloor-hosted hydrothermal systems, including the: (i) the reaction zone, where peak pressure and temperature conditions are encountered near the base of the near-axis hydrothermal system; (ii) the upflow zone, where ascending hydrothermal fluids react with the oceanic crust; and (iii) the mixing zone, where hydrothermal vent fluids are emitted into ambient seawater. We focused models using nominal parameter values discussed by Tosca and Tutolo (*15*). For reaction zone conditions, these include rock-buffered fluid chemistry in the Na_2_O-K_2_O-CaO-MgO-FeO-Fe_2_O_3_-Al_2_O_3_-SiO_2_-H_2_O-HCl-H_2_S system in the presence of plagioclase solid solution, epidote solid solution, clinochlore, K-feldspar, quartz, fayalite, pyrrhotite, magnetite, and aqueous fluid at 400°C and 400 bar at pH 5.0.

**Fig. 9.**
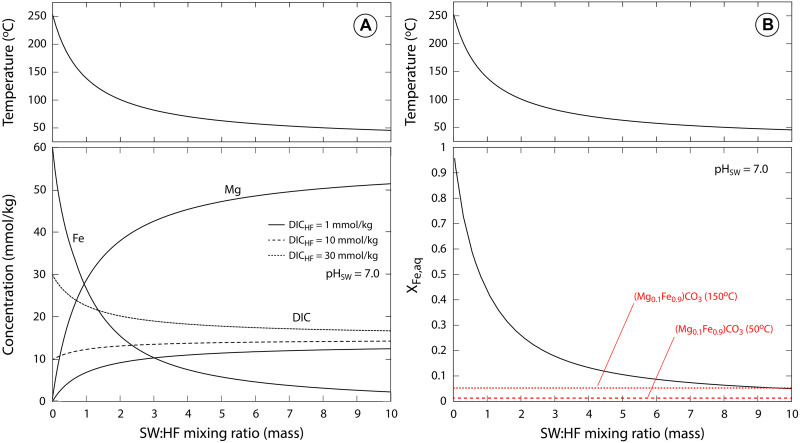
Reaction path model of aqueous Fe and Mg concentrations during mixing between hydrothermal vents and anoxic, low-SO_4_ seawater. Calculated fluid composition (**A**) and activity fraction of Fe (X_Fe,aq_; **B**) derived from the mixing between high Fe/H_2_S fluids (at 250°C and 250 bar) and anoxic SO_4_-free seawater (at pH 7 and 15 mmol/kg of DIC) as a function of DIC concentration in the hydrothermal vent fluid (DIC_HF_). Siderite (Sid) was suppressed from the calculation. Red dashed lines in (B) indicate thresholds above which appreciable Mg-bearing (Fe,Mg)CO_3_ solids (i.e., greater than 10 mol % Mg) are thermodynamically unstable relative to more Fe-rich compositions. These compositions are stabilized only at very high sewater:hydrothermal fluid mixing ratios (taking into account decreases in temperature with increased mixing). SW, seawater; HF, hydrothermal fluid.

We then prescribed a cooling and decompression pathway to simulate the release of these fluids from the reaction zone and their upflow through the oceanic crust. This included interaction with fresh basalt to simulate continued interaction with wall rock at a fluid/rock mass ratio of 750. Last, the fluids were mixed with anoxic cold (4°C) seawater at pH 7 and [DIC] = 15 mmol/kg (Fig. 9). In these final mixing calculations, we examined the effect of variable [DIC] in the hydrothermal vent fluids. To examine the effects of these interactions on the stability of compositions in the (Fe,Mg)CO_3_ solid solution, we suppressed siderite from the mixing calculations and monitored the aqueous activity fraction of Fe relative to Mg, X_Fe,aq_, as a quantitative indication of which compositions may be stable across hydrothermal vent fluid–seawater mixing. Together, these calculations show that (Fe,Mg)CO_3_ solids with appreciable Mg content (i.e., 10 mol % and higher) are stabilized only by fluid compositions that are dominated by seawater.

The combination of thermodynamic constraints from geochemical modeling and petrographic relationships implies that Mg-bearing rims of larger microcrystals and magnesian rhombs and aggregates indicate an origin from fluids dominated by seawater. The association of siderite microcrystals with nanoparticulate greenalite and apatite, previously interpreted to be precipitates from proximal and distal vent plumes (*25*, *38*, *39*), provides additional support for an origin from mixing between vent fluid and seawater.

Last, reaction path modeling of the hydrothermal alteration of basalt by oxygen- and sulfate-free seawater under mid-ocean–ridge conditions predicts that Fe^2+^ emitted into seawater precipitates as pyrite, greenalite, and possibly siderite, depending on kinetic barriers (*15*). In addition, modeling suggests that P may also be liberated from seafloor basalts by hydrothermal fluids and precipitated as fluorapatite upon mixing with seawater (*25*). The agreement between predictions from theoretical models and nanoscale observations from Paleoarchean ferruginous cherts provides strong evidence for an abiotic hydrothermal origin for the siderite microcrystals. The paragenetic sequence in the cherts, with apatite nanoparticles enclosed in siderite, is consistent with the predicted sequence of plume particle precipitation: pyrite, greenalite, and apatite and lastly siderite (*15*, *25*).

### Implications for the origin of siderite in iron formations

**Fig. 10.**
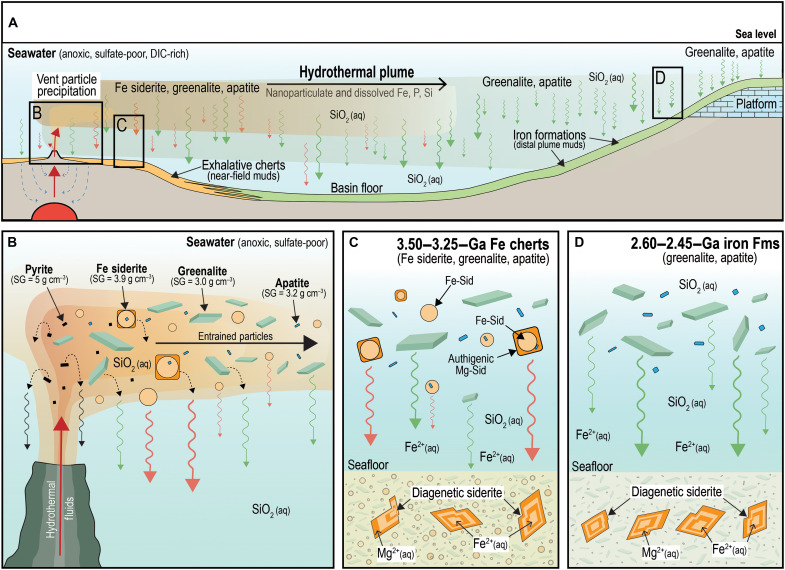
Proposed model for the deposition of vent-proximal Fe cherts and vent-distal BIFs. (**A**) Hydrothermal venting of Fe^2+^ and PO_4_^3−^, and mixing with anoxic, sulfate-free seawater, producing plumes of particulate siderite (Sid), greenalite (Gre), and apatite (Ap). (**B**) Formation, transportation, and gravitational settling of Sid, pyrite, Gre, and Ap particles in plumes around vents. SG, specific gravity. (**C**) Close to seafloor vents, Sid microcrystals settle from the plume with Gre and Ap nanoparticles, forming uncompacted chemical muds with 70 to 90% porosity. Diagenetic Mg-rich Sid rhombs form in the sediment. (**D**) Far from seafloor vents, plume particles comprise mainly Gre and Ap nanoparticles. The lack of Sid plume particles in BIFs may reflect lower seawater DIC in Neoarchean oceans and/or preferential gravitational settling. In hydrothermal sediments, Sid is diagenetic, forming compositionally zoned aggregates of Mg-rich Sid.

Siderite is one of the most abundant Fe phases in well-preserved BIFs such as those of the Hamersley and Transvaal basins (*3*). In these rocks, it typically occurs as 10- to 30-μm-long Mg-rich rhombs and aggregates concentrated along bedding planes (Fig. 7A). The size, shape, and composition suggest that siderite in these BIFs was not deposited from the water column. In the Hamersley-Transvaal BIFs, Fe-rich siderite microcrystals are rare or absent from laminated chert containing greenalite and apatite nanoparticles (Fig. 7, C to H), which are interpreted to have been deposited from distal vent plumes (*15*, *29*, *38*, *39*, *48*). Most younger BIFs (<2.60 Ga) are preserved within predominantly sedimentary, rather than volcanic, successions, and the absence of Fe-siderite plume particles in them implies that either they did not form during hydrothermal venting after the Paleoarchean or, if they did form, they were not deposited and preserved with greenalite and apatite particles. If the latter, then one possibility is that siderite plume particles settled from the water column before reaching the BIF depocenter (Fig. 10) due to its higher specific gravity relative to greenalite and apatite.

Geochemical reaction path models of mixing between hydrothermal vent fluids and seawater indicate that the concentration of DIC in seawater is one of the strongest controls on the abundance of siderite produced during mixing. Modeling results indicate that seawater DIC exerts far more control on siderite abundance than the concentration of DIC in the hydrothermal fluid [Fig. 6 in (*15*)]. The reasons for this stem from the relationship between seawater DIC and buffering capacity (or the resistance of seawater to perturbations in pH). When seawater DIC is relatively high, the concomitant change in pH induced on mixing with acidic hydrothermal vent fluids is lower, therefore preserving a higher pH, DIC, and alkalinity (*15*). This results in higher levels of supersaturation maintained across a broader portion of the mixing profile, which, in turn, generates more siderite in the mineral assemblage. Conversely, as seawater DIC decreases, pH is more severely affected on vent fluid mixing (i.e., it decreases significantly), which leads to sharp decreases in siderite saturation. These results imply that a first-order geochemical control on depositional siderite abundances in Paleoarchean BIFs versus younger counterparts may relate to progressive decreases in seawater DIC.

A diagenetic origin for siderite in these vent-distal BIFs is consistent with petrographic and experimental evidence. Several pathways for postdepositional siderite growth are possible, including abiotic precipitation in sediment pores enriched in dissolved Fe^2+^ from ferruginous seawater and bacterial reduction of Fe(III) oxides/hydroxides by DIR in areas of high biological productivity. The latter requires that dissolved Fe^2+^ is first oxidized in the water column by photoautotrophs and the resulting Fe(III) oxides/hydroxides are then codeposited with the organic debris of the photosynthesizers. In this case, the depleted δ^13^C-isotopic composition of the siderite is interpreted to reflect mixing between isotopically light organic carbon (−30‰) with seawater DIC (near-zero per mil) in pore waters. This process, which suggests high biological productivity in the water column, is viewed by some as unlikely because the total organic carbon (TOC) content of BIFs and Fe cherts is exceedingly low (*49*). Instead, the low TOC contents of BIFs may reflect low primary productivity in oceans where high rates of chemical sedimentation diluted organic matter in the precursor sediments of BIF (*50*).

An alternative pathway for siderite growth is abiotic precipitation in sediment porewaters. This interpretation implies that seawater was enriched in dissolved Fe^2+^ and that there was a steady supply of Fe^2+^ during crystal growth, possibly replenished from ferruginous seawater and/or compactional dewatering. The range of depleted δ^13^C compositions of siderite may reflect carbon kinetic isotope effects associated with abiotic siderite growth from supersaturated solutions with a bottom and/or pore water DIC reservoir with near-zero δ^13^C values (*14*). The abiotic precipitation of siderite in sediment porewaters can explain the texture, abundance, and composition of much of the siderite in Hamersley-Transvaal BIFs (*14*).

### Hydrothermal Fe(II)-rich sediments on wet rocky planets and satellites

Our results from Paleoarchean exhalative Fe cherts align with reaction path modeling predicting that mixing between hydrothermal fluids and anoxic, sulfate-free seawater, enriched in DIC, SiO_2_(aq) and Fe^2+^(aq), produces pyrite, greenalite, siderite, and apatite (*15*, *25*). The primary mineralogy of Paleoarchean Fe cherts may have implications for the origin of younger BIFs, which are widely considered to have been sourced from distal vent plumes. We argue that the mixing of seawater and effluent from vents at elevated temperatures resulted in the formation of particulate ferrous-rich phases and Ca phosphate that were dispersed across the ocean in diffuse plumes and deposited in basin floor, slope, and shelf environments during major marine incursions.

The absence of microcrystalline siderite from greenalite- and apatite-bearing chert in BIFs suggests that siderite was not deposited from distal hydrothermal plumes but grew as larger crystals along bedding planes from marine porewaters. The abundance of diagenetic siderite in the thick and laterally extensive BIFs of the Hamersley and Transvaal basins implies a vast reservoir of dissolved Fe^2+^ and DIC in sediment porewaters during siderite growth. The lack of diagenetic siderite in underlying siliciclastic shales (e.g., Mt. McRae Shale, Hamersley Group; Jeerinah Formation, Fortescue Group) and/or carbonates (Campbellrand Subgroup, Transvaal Supergroup; Wittenoom Formation, Hamersley Group) suggests that siderite was not a universal authigenic mineral in Archean marine sediments. The co-occurrence of abundant siderite with iron formations and Fe-rich cherts suggests that conditions for ferrous carbonate precipitation were linked to the episodic incursion of hydrothermal-seawater mixtures enriched in dissolved Fe(II), P, and SiO_2_.

The findings add to a growing body of observational and geochemical data, which is prompting a rethink about the origin of iron formations. In contrast to traditional models, which favor biologically mediated oxidation of ferrous iron in the surface ocean followed by microbial iron-oxide reduction (*12*), our results suggest that most of the iron was deposited as ferrous-rich phases that formed from Fe(II) liberated during seawater alteration of basaltic crust. Rather than being restricted to zones of coastal upwelling with high biological productivity, iron deposition may have occurred over large areas of the seafloor via settling from hydrothermal plumes, which acted as marine conveyor belts transporting vent-derived nanoparticulate and dissolved Fe(II). According to this model, iron formations or jaspilites were not biologically deposited chemical sediments and therefore potential indicators of early Archean photosynthesis, but abiogenic products of mixing between hydrothermal vent fluids and anoxic seawater. Given the likely abundance of water-rich rocky planets and satellites with basaltic crust beyond Earth, our observations from the Paleoarchean rock record and geochemical modeling suggest that hydrothermal sediments comprising Fe(II)-rich (pyrite, greenalite, and siderite) and P-rich (apatite) nanoparticles are likely ubiquitous on volcanically and hydrothermally active planets covered in oceans or shallow seas.

## MATERIALS AND METHODS

### Optical and SEM

Samples of ferruginous chert were cut and used to prepare polished thin sections. Polished thin sections were initially examined using a Nikon ECLIPSE Polarizing Microscope to collect mineralogical and textural information. Following optical microscopy, polished thin sections were characterized with backscattered electron imaging using an FEI Verios (field-emission source) SEM located in the Centre for Microscopy, Characterisation and Analysis (CMCA) at the University of Western Australia (UWA). The SEM was fitted with an Oxford Instruments XMax EDS, which was used for qualitative chemical analysis of mineral grains using the AZtec software from Oxford Instruments.

### Electron probe microanalysis

The element distributions in the siderite grains were investigated by point analyses and x-ray maps generated using WDS. Points and maps for major and minor elements (Sr La TAPL, Mg Ka TAPL, Ca Ka PETL, Mn Ka LIFL, and Fe Ka LIFL) were collected on a JEOL 8530F Hyperprobe Plus equipped with five WD spectrometers at 15-kV accelerating voltage and 10-nA (points) and 25-nA (maps) beam currents. An analysis file was created using the Probe for EPMA software from Probe Software with natural carbonate standard reference materials. Background values were calculated using a Mean Atomic Number correction. The x-ray maps were collected using Probe Image from Probe Software, with 100-ms dwell time per pixel and pixel size of 0.25 μm by 0.25 μm. The map data were processed using Calc Image from Probe Software to generate quantitative distribution maps for each element.

### Focussed ion beam

Lamellae for TEM analyses were cut from polished thin sections. FIB techniques were used to prepare ~100-nm-thick TEM lamellae using an FEI Helios NanoLab G3 CX DualBeam instrument located at CMCA, UWA. The areas selected for TEM analysis were first coated with a strip of Pt, 2 μm thick to protect the surface, and then trenches 7 μm deep were milled on either side of the strip using a Ga ion beam with 30-kV voltage and 9.3-nA current. The lamellae were then cut away from the samples and welded to Cu TEM grids. The lamellae were thinned with the Ga ion beam at 30 kV and 0.79 and 0.23 nA before cleaning at 5 kV and 41 pA and polishing at 2 kV and 23 pA.

### Transmission electron microscopy

TEM data were collected (at 200 kV) using an FEI Titan G2 80-200 TEM/STEM instrument with ChemiSTEM technology in CMCA at UWA. Images collected using high-resolution TEM, bright-field TEM and STEM, and high-angle annular dark-field (HAADF) STEM modes were processed using TEM Imaging and Analysis software from FEI. Qualitative EDS maps and spectra were collected with an FEI Super-X EDS detector and processed using the Esprit software (Bruker).

### Thermodynamic data and geochemical modeling

Thermodynamic calculations in the FeCO_3_-MgCO_3_-H_2_O system used enthalpy of mixing data for the (Fe,Mg)CO_3_ solid solution determined by Chai and Navrotsky (*46*) and the assumption of a regular solid solution model characterized by ideal entropy of mixing (Supplementary Materials). These data, along with solubility determinations of the FeCO_3_ and MgCO_3_ endmembers as a function of temperature, allow aqueous solution–solid solution thermodynamic calculations as a function of temperature following the theoretical framework described by Glynn (*51*) (Supplementary Materials). To examine the stability of compositions in the (Fe,Mg)CO_3_ solid solution during the mixing between Archean hydrothermal vent fluids and ambient seawater, we estimated equilibrium solution compositions and thermodynamically predicted mineralogy by executing reaction path models.

## Supplementary Materials

### This PDF file includes:

Supplementary Text Figs. S1 to S3 References
