# Supplementary material for: Siderite precipitation in Paleoarchean oceans during hydrothermal venting
Source: Sci Adv. 2025 Nov 12;11(46):eady6851. doi: 10.1126/sciadv.ady6851 (PMC12609112; doi:10.1126/sciadv.ady6851)
Supplement: Supplementary file 1 — Supplementary Text Figs. S1 to S3 References [file sciadv.ady6851_sm.pdf]

Science Advances
AAAS

Supplementary Materials for

Siderite precipitation in Paleoarchean oceans during hydrothermal venting

Birger Rasmussen *et al.*

Corresponding author: Birger Rasmussen, birger.rasmussen@uwa.edu.au

Sci. Adv. **11**, eady6851 (2025)
DOI: 10.1126/sciadv.ady6851

This PDF file includes:

Supplementary Text
Figs. S1 to S3
References

Supplementary Materials

Thermodynamics of the (Fe,Mg)CO_3 solid solution

Solid solutions along the magnesite-siderite join were synthesized by Chai and Navrotsky (*46*) and used to determine the enthalpy of mixing through calorimetry. These analyses showed that the (Fe,Mg)CO_3 solid solution is characterised by a small positive and symmetric enthalpy of mixing. These characteristics, along with a volume of mixing very close to zero, are consistent with a regular solution model with:

$$\Delta H_{mix} = W X_{Fe} X_{Mg}$$

where W is the interaction parameter, and X_{Fe} and X_{Mg} are the mole fractions of Fe^{2+} and Mg^{2+} in the solid solution, respectively. Chai and Navrotsky's (*46*) data yielded a value for W = 4.44 ± 0.75 kJ/mol, in excellent agreement with empirical relationships between volume mismatch and interaction parameters for several solid solutions.

These data also allow the calculation of the Gibbs free energy of mixing, assuming a regular solution model and ideal entropy of mixing. This in turn allows quantification of the solvus and spinodal curves in the (Fe,Mg)CO_3 system (**Fig. S1**) and the critical temperature (T_c), which corresponds to the lowest possible temperature at which a complete solid solution may form:

$$T_c = \frac{W}{2R} = 267\ K$$

where R is the molar gas constant. These results show that complete solid solution in (Fe,Mg)CO_3 is predicted over a wide range in temperature (i.e., above -6°C) (**Fig. S1**).

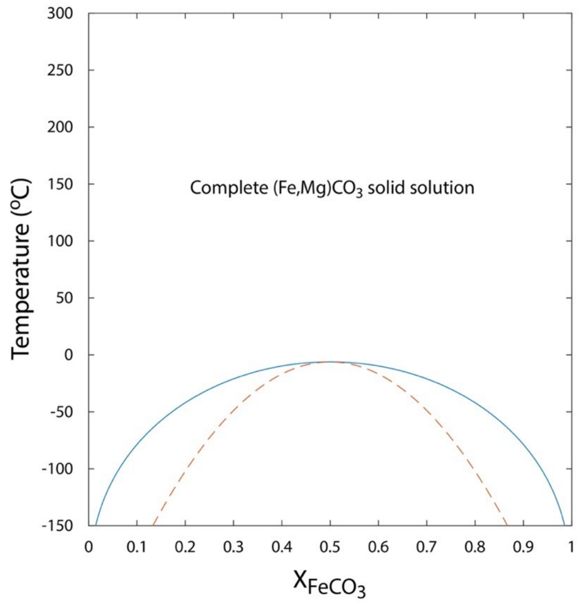

Fig. S1. Phase diagram for the (Fe,Mg)CO_3 system as a function of mole fraction of $FeCO_3$ (X_{FeCO3}) (after ref. *46*). The solid curve corresponds to the solvus curve, which demarcates the miscibility gap, and the dashed curve corresponds to the spinodal curve, below which spinodal decomposition becomes possible.

Solid solution-aqueous solution thermodynamics

Thermodynamic data published by Chai and Navrotsky (*46*), in addition to solubility measurements of endmember $FeCO_3$ (siderite) and $MgCO_3$ (magnesite) (*52, 53*), permit analysis of equilibrium conditions between aqueous solutions and $(Fe,Mg)CO_3$ phases as a function of composition and temperature.

For the binary $(Fe,Mg)CO_3$ solid solution, application of the law of mass action to the solubility product reactions of the endmember components yields:

$$a_{Fe2+}a_{CO32-} = K_{FeCO3}a_{FeCO3} = K_{FeCO3}X_{FeCO3}\gamma_{FeCO3}$$
$$a_{Mg2+}a_{CO32-} = K_{MgCO3}a_{MgCO3} = K_{MgCO3}X_{MgCO3}\gamma_{MgCO3}$$

Where a_{Fe2+}, a_{Mg2+}, a_{CO32-} are the activities of Fe^{2+}, Mg^{2+} and CO_3^{2-} in aqueous solution, and a_{FeCO3}, a_{MgCO3}, X_{FeCO3}, X_{MgCO3}, γ_{FeCO3}, and γ_{MgCO3} are the activities, mole fractions, and activity coefficients, respectively, of components $FeCO_3$ and $MgCO_3$ in the equilibrium solid solution. K_{FeCO3} and K_{MgCO3} are the solubility products of pure siderite and magnesite, respectively.

In solid solution systems, it becomes convenient to introduce the variable $\Sigma\Pi$ (*51*), which expresses the additive effects of the two components on the solubility product constant of the solid solution (thus defining the "solidus" curve):

$$\Sigma\Pi_{\text{eq}} = K_{FeCO3}X_{FeCO3}\gamma_{FeCO3} + K_{MgCO3}X_{MgCO3}\gamma_{FeCO3}$$

where $\Sigma\Pi_{\text{eq}}$ is the value of the $\Sigma\Pi$ variable at equilibrium.
The corresponding ion activities present in an aqueous solution in equilibrium with a binary solid solution (which defines the "solutus" curve) of a given composition are then given by:

$$\Sigma\Pi_{\text{eq}} = 1 \Bigg/ \left(\frac{X_{Fe,aq}}{K_{FeCO3}\gamma_{FeCO3}} + \frac{X_{Mg,aq}}{K_{MgCO3}\gamma_{MgCO3}}\right)$$

Where the aqueous activity fractions $X_{Fe,aq}$ and $X_{Mg,aq}$ are defined as:

$$X_{Fe,aq} = \frac{a_{Fe2+}}{(a_{Fe2+} + a_{Mg2+})}$$
$$X_{Mg,aq} = \frac{a_{Mg2+}}{(a_{Fe2+} + a_{Mg2+})}$$

Activity coefficients of the solid solution are derived from Guggenheim's polynomial expansion of the excess Gibbs free energy of mixing (*54*):

$$\ln \gamma_{MgCO3} = X_{FeCO3}^2[a_0 - a_1(3X_{MgCO3} - X_{FeCO3}) + a_2(X_{MgCO3} - X_{FeCO3})(5X_{MgCO3} - X_{FeCO3}) + \ldots]$$
$$\ln \gamma_{FeCO3} = X_{MgCO3}^2[a_0 + a_1(3X_{FeCO3} - X_{MgCO3}) + a_2(X_{FeCO3} - X_{MgCO3})(5X_{FeCO3} - X_{MgCO3}) + \ldots]$$

Given the small difference in the relative sizes of the substituting ions, and analytical data consistent with a regular solid solution model (*46*), our treatment includes only the first terms of

the excess Gibbs free energy of mixing functions (*54*), and we obtain the parameter a_0 through the following relation:

$$a_0 = \frac{2T_c}{T}$$

Solid solution-aqueous solution data

Relatively few experimental data demonstrating equilibrium between solid solution and aqueous solution are available for $(Fe,Mg)CO_3$ as a function of temperature. However, Johannes (*47*) conducted experiments between 200ºC and 500ºC and 1000 bar in the Mg-Fe-CO_3-Cl-H_2O system and reported detailed experimental procedures, final solid compositions and final aqueous solution compositions, though provided no explicit demonstration that thermodynamic equilibrium had been obtained. Interpretation of Johannes' (*47*) data in the framework above requires estimation of ion activities under the experimental conditions. To permit these calculations, we calculated appropriate thermodynamic data for the aqueous Mg-Fe-CO_3-Cl-H_2O system across a range of temperatures and at 1000 bar using the Python module pyGCC (*55*), which uses the revised HKF equation of state to calculate the apparent standard molal Gibbs free energies of aqueous solutes at elevated temperature and pressure (*56*). These data were then used to perform speciation and ion activity coefficient calculations (implemented in the React module of Geochemists Workbench; Bethke et al. [*57*]) of experiments at 200ºC and 250ºC, yielding the data displayed on **Fig. S2**.

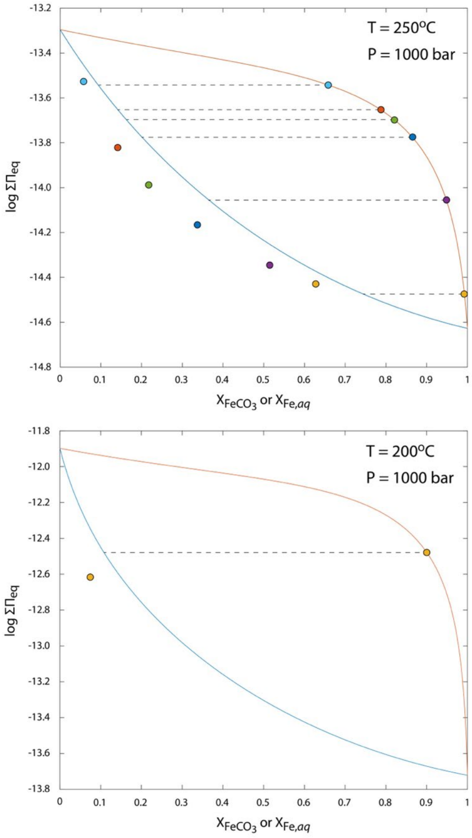

Fig. S2. Solid solution-aqueous solution equilibria for $(Fe,Mg)CO_3$ solids at 250ºC (top) and 200ºC (bottom) and 1000 bar. The "solidus" curve, shown in red, indicates the thermodynamic solubility of $(Fe,Mg)CO_3$ solids as a function of mole fraction of $FeCO_3$ (X_{FeCO3}). The "solutus" curve, shown in blue,

indicates coexisting aqueous solution composition (in terms of the activity fraction of Fe relative to Mg, or X_{Feaq}) in equilibrium with a solid of a given composition. Data points for solid and aqueous solution compositions are from Johannes (*47*), with X_{Feaq} calculated using procedures described in the supplemental material. Dashed lines indicate tie lines between co-existing solid and aqueous solution compositions, with data points from the same experiment denoted in the same colors.

Reaction path calculations

To examine the stability of compositions in the (Fe,Mg)CO_3 solid solution during the mixing between Archean hydrothermal vent fluids and ambient seawater, we estimated equilibrium solution compositions and thermodynamically predicted mineralogy by executing reaction path models for subseafloor-hosted hydrothermal systems (see ref. *15*). We focused models using nominal parameter values discussed by Tosca and Tutolo (*15*). For reaction zone conditions, these include rock-buffered fluid chemistry in the Na_2O-K_2O-CaO-MgO-FeO-Fe_2O_3-Al_2O_3-SiO_2-H_2O-HCl-H_2S system in the presence of plagioclase solid solution, epidote solid solution, clinochlore, K-feldspar, quartz, fayalite, pyrrhotite, magnetite, and aqueous fluid at 400ºC and 400 bar at pH 5.0.

We then prescribed a cooling and decompression pathway to simulate the release of these fluids from the reaction zone and their upflow through the oceanic crust. This included interaction with fresh basalt to simulate continued interaction with wall rock at a fluid/rock mass ratio of 750. Finally, the fluids were mixed with anoxic cold (4ºC) seawater at pH 7 and [DIC] = 15 mmol/kg (**Fig. S3**). In these final mixing calculations, we examined the effect of variable [DIC] in the hydrothermal vent fluids. In order to examine the effects of these interactions on the stability of compositions in the (Fe,Mg)CO_3 solid solution, we suppressed siderite from the mixing calculations and monitored the aqueous activity fraction, $X_{Fe,aq}$ as a quantitative indication of which compositions may be stable across hydrothermal vent fluid-seawater mixing.

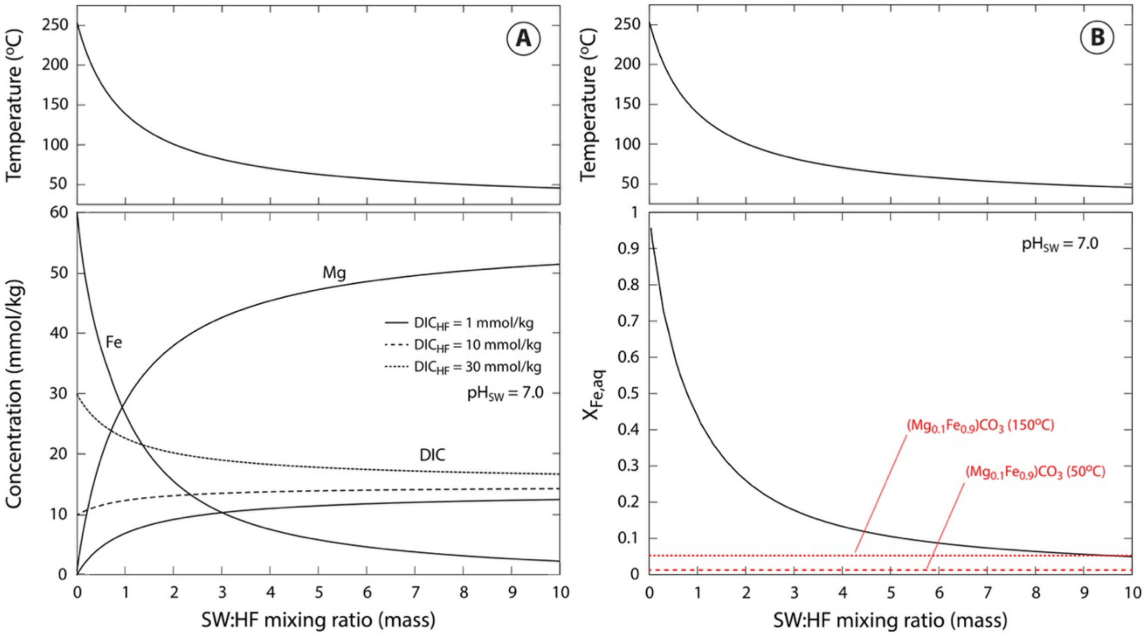

Fig. S3. Calculated fluid composition (A) and activity fraction of Fe ($X_{Fe,aq}$; B) derived from the mixing between high Fe/H_2S fluids (at 250ºC and 250 bar) and anoxic SO_4-free seawater (at pH 7 and 15 mmol/kg DIC) as a function of DIC concentration in the hydrothermal vent fluid (DIC_{HF}). Siderite was suppressed from the calculation. Red dashed lines in (B) indicate thresholds above which appreciable Mg-bearing (Fe,Mg)CO_3 solids (i.e., greater than 10 mol % Mg) are thermodynamically unstable relative to more Fe-

rich compositions. These compositions are stabilized only at very high SW:HF mixing ratios (taking into account decreases in temperature with increased missing).

REFERENCES AND NOTES

1. H. Ohmoto, Y. Watanabe, K. Kumazawa, Evidence from massive siderite beds for a CO_2-rich atmosphere before ~1.8 billion years ago. *Nature* **429**, 395–399 (2004).

2. D. E. Ayres, Genesis of iron-bearing minerals in the Brockman Iron Formation mesobands in the Dales Gorge Member, Hamersley Group, Western Australia. *Econ. Geol.* **67**, 1214–1233 (1972).

3. C. Klein, N. J. Beukes, Geochemistry and sedimentology of a facies transition from limestone to iron-formation deposition in the early Proterozoic Transvaal Supergroup, South Africa. *Econ. Geol.* **84**, 1733–1774 (1989).

4. N. J. Beukes, C. Klein, A. J. Kaufman, J. M. Hayes, Carbonate petrography, kerogen distribution, and carbon and oxygen isotopic variations in an early Proterozoic transition from limestone to iron-formation deposition, Transvaal Supergroup, South Africa. *Econ. Geol.* **85**, 663–690 (1990).

5. J. Kaufman, J. M. Hayes, C. Klein, Primary and diagenetic controls of isotopic compositions of iron-formation carbonates. *Geochim. Cosmochim. Acta* **54**, 3461–3473 (1990).

6. M. M. Tice, D. R. Lowe, Photosynthetic mats in the 3,416-Myr-old ocean. *Nature* **431**, 549–552 (2004).

7. R. Bolhar, M. J. Van Kranendonk, B. S. Kamber, A trace element study of siderite-jasper banded iron formation in the 3.45 Ga Warrawoona Group, Pilbara Craton—Formation from hydrothermal fluids and shallow seawater. *Precambrian Res.* **137**, 93–114 (2005).

8. F. Gäb, C. Ballhaus, J. Siemens, A. Heuser, M. Lissner, T. Geisler, D. Garbe-Schönberg, Siderite cannot be used as CO_2 sensor for Archaean atmospheres. *Geochim. Cosmochim. Acta* **214**, 209–225 (2017).

9. C. Z. Jiang, N. J. Tosca, Fe(II)-carbonate precipitation kinetics and the chemistry of anoxic ferruginous seawater. *Earth Planet. Sci. Lett.* **506**, 231–242 (2019).

10. C. Z. Jiang, N. J. Tosca, Growth kinetics of siderite at 298.15 K and 1 bar. *Geochim. Cosmochim. Acta* **274**, 97–117 (2020).

11. J. C. G. Walker, Suboxic diagenesis in banded iron formations. *Nature* **309**, 340–342 (1984).

12. K. Konhauser, N. Planavsky, D. Hardisty, L. Robbins, T. Warchola, R. Haugaard, S. Lalonde, C. Partin, P. Oonk, H. Tsikos, T. Lyons, A. Bekker, C. Johnson, Iron formations: A global record of Neoarchaean to Palaeoproterozoic environmental history. *Earth Sci. Rev.* **172**, 140–177 (2017).

13. N. J. Beukes, J. Gutzmer, Origin and paleoenvironmental significance of major iron formations at the Archean-Paleoproterozoic boundary. *Rev. Econ. Geol.* **15**, 5–47 (2008).

14. C. Z. Jiang, I. Halevy, N. J. Tosca, Kinetic isotope effect in siderite growth: Implications for the origin of banded iron formation siderite. *Geochim. Cosmochim. Acta* **322**, 260–273. (2022).

15. N. J. Tosca, B. M. Tutolo, Hydrothermal vent fluid-seawater mixing and the origins of Archean iron formation. *Geochim. Cosmochim. Acta* **352**, 51–68 (2023).

16. H. Hickman, *East Pilbara Craton: A Record of One Billion Years in the Growth of Archean Continental Crust* (Geological Survey of Western Australia), 2021).

17. R. Buick, “Life and conditions in the early Archaean: Evidence from 3500 m.y. old shallow-water sediments in the Warrawoona Group, North Pole, Western Australia,” thesis, University of Western Australia (1985).

18. T. Djokic, M. J. Van Kranendonk, K. A. Campbell, M. R. Walter, C. R. Ward, Earliest signs of life on land preserved in ca. 3.5 Ga hot spring deposits. *Nat. Comm.* **8**, 15263 (2017).

19. W. Nijman, K. De Bruin, M. Valkering, Growth fault control of early Archaean cherts, barite mounds, and chert-barite veins, North Pole Dome, Eastern Pilbara, Western Australia. *Precambrian Res.* **88**, 25–52 (1999).

20. J. S. R. Dunlop, M. D. Muir, V. A. Milne, D. I. Groves, A new microfossil assemblage from the Archaean of Western Australia. *Nature* **274**, 676–678 (1978).

21. M. R. Walter, R. Buick, J. S. R. Dunlop, Stromatolites 3,400–3,500 Myr old from the North Pole area, Western Australia. *Nature* **284**, 443–445 (1980).

22. S. M. Awramik, J. W. Schopf, M. R. Walter, Filamentous fossil bacteria from the Archean of Western Australia. *Precambrian Res.* **20**, 357–374 (1983).

23. C. M. Johnson, Y.-Y. Zheng, T. Djokic, M. J. Van Kranendonk, A. D. Czaja, E. E. Roden, B. L. Beard, Early Archean biogeochemical iron cycling and nutrient availability: New insights from a 3.5 Ga land-sea transition. *Earth Sci. Rev.* **228**, 103992 (2022a).

24. C. M. Johnson, Y.-Y. Zheng, T. Djokic, M. J. Van Kranendonk, A. D. Czaja, E. E. Roden, B. L. Beard, Reply to comment by Birger Rasmussen and Janet R. Muhling on “Early Archean biogeochemical iron cycling and nutrient availability: New insights from a 3.5 Ga land-sea transition” by Johnson et al. *Earth Sci. Rev.* **231**, 104087 (2022).

25. B. Rasmussen, J. R. Muhling, N. J. Tosca, Nanoparticulate apatite and greenalite in oldest well-preserved hydrothermal vent precipitates. *Sci. Adv.* **10**, eadj4789 (2024a).

26. M. Hoashi, D. C. Bevacqua, T. Otake, Y. Watanabe, A. H. Hickman, S. Utsunomiya, H. Ohmoto, Primary haematite formation in an oxygenated sea 3.46 billion years ago. *Nat. Geosci.* **2**, 301–306 (2009).

27. B. Rasmussen, B. Krapez, J. R. Muhling, Hematite replacement of iron-bearing precursor sediments in the 3.46-b.y.-old Marble Bar Chert, Pilbara Craton, Australia. *Geol. Soc. Am. Bull.* **126**, 1245–1258 (2014b).

28. B. Rasmussen, J. R. Muhling, Comment on “Early Archean biogeochemical iron cycling and nutrient availability: New insights from a 3.5 Ga land-sea transition” by Clark M. Johnson, Xin-Yuan Zheng, Tara Djokic, Martin J. Van Kranendonk, Andrew D. Czaja, Eric E. Roden, Brian L. Beard, 2022, Earth-Science Reviews. *Earth Sci. Rev.* **231**, 104088 (2022).

29. J. R. Muhling, B. Rasmussen, Widespread deposition of greenalite to form banded iron formations before the Great Oxidation Event. *Precambrian Res.* **339**, 105619 (2020).

30. A. C. Allwood, M. R. Walter, B. S. Kamber, C. P. Marshall, I. W. Burch, Stromatolite reef from the Early Archaean era of Australia. *Nature* **441**, 714–718 (2006).

31. A. C. Allwood, I. Burch, M. R. Walter, Stratigraphy and facies of the 3.43 Ga Strelley Pool Chert in the southwestern North Pole Dome, Pilbara Craton, Western Australia. *West Australia Geol. Surv. Rec.* **11**, 22 (2007).

32. D. R. Lowe, Restricted shallow-water sedimentation of Early Archean stromatolitic and evaporitic strata of the Strelley Pool Chert, Pilbara Block, Western Australia. *Precambrian Res.* **19**, 239–283 (1983).

33. B. Rasmussen, J. R. Muhling, A. Sadekov, Snapshot of a Paleoarchean seafloor: Evidence from 3.43-3.35 Ga Strelley Pool chert-pebble conglomerate for deposition, silicification and erosion of hydrothermal greenalite-apatite precipitates. *Precambrian Res.* **412,** 107531 (2024)

34. S. Guggenheim, R. A. Eggleton, Modulated crystal structures of greenalite and caryopilite: A system with long-range, in-plane structural disorder in the tetrahedra sheet. *Can. Mineral.* **36**, 163–179 (1998).

35. B. Rasmussen, B. Krapez, J. R. Muhling, A. A. Suvorova, Precipitation of iron silicate nanoparticles in early Precambrian oceans marks Earth's first iron age. *Geology* **43**, 303–306 (2015).

36. E. Spencer, F. G. Percival, The structure and origin of the banded hematite jaspers of Singhbhum, India. *Econ. Geol.* **47**, 365–383 (1952).

37. B. Rasmussen, B. Krapez, D. B. Meier, Replacement origin for hematite in 2.5 Ga banded iron formation: Evidence for postdepositional oxidation of iron-bearing minerals. *Geol. Soc. Am. Bull.* **126**, 438–446 (2014).

38. B. Rasmussen, J. R. Muhling, B. Krapez, Greenalite and its role in the genesis of early Precambrian iron formations – A review. *Earth Sci. Rev.* **217**, 103613 (2021a).

39. B. Rasmussen, J. R. Muhling, A. Suvorova, W. W. Fischer, Apatite nanoparticles in 3.46–2.46 Ga iron formations: Evidence for phosphorus-rich hydrothermal plumes on early Earth. *Geology* **49**, 647–651 (2021).

40. N. J. Beukes, Early options in photosynthesis. *Nature* **431**, 522–523 (2004).

41. M. J. Van Kranendonk, G. E. Webb, B. S. Kamber, Geological and trace element evidence for a marine sedimentary environment of deposition and biogenicity of 3.45 Ga stromatolitic carbonates in the Pilbara Craton, and support for a reducing Archaean ocean. *Geobiology* **1**, 91–108 (2003).

42. Y. Kato, K. Suzuki, K. Nakamura, A. H. Hickman, M. Nedachi, M. Kusakabe, D. C. Bevacqua, H. Ohmoto, Hematite formation by oxygenated groundwater more than 2.76 billion years ago. *Earth Planet. Sci. Lett.* **278**, 40–49 (2009).

43. J. N. Fitzsimmons, S. G. John, C. M. Marsay, C. L. Hoffman, S. L. Nicholas, B. M. Toner, C. R. German, R. M. Sherrell, Iron persistence in a distal hydrothermal plume supported by dissolved–particulate exchange. *Nat. Geosci.* **10**, 195–201 (2017).

44. C. R. German, K. L. Von Damm, Hydrothermal processes. *Treatise Geochem.* **6**, 181–222 (2006).

45. M. K. Tivey, Generation of seafloor hydrothermal vent fluids and associated mineral deposits. *Oceanography* **20**, 50–65 (2007).

46. L. Chai, A. Navrotsky, Synthesis, characterization, and enthalpy of mixing of the $(Fe,Mg)CO_3$ solid solution. *Geochim. Cosmochim. Acta* **60**, 4377–4383 (1996).

47. W. Johannes, Siderit-Magnesit-Mischkristallbildung im system Mg^{2+}-Fe^{2+}-CO_3^{2-}-Cl_2−H_2O. *Contrib. Mineral. Petrol.* **21**, 311–318 (1969).

48. B. Rasmussen, J. R. Muhling, A. Suvorova, B. Krapez, Greenalite precipitation linked to the deposition of banded iron formations downslope from a late Archaean carbonate platform. *Precambrian Res.* **290**, 49–62 (2017).

49. H. D. Holland, Sedimentary mineral deposits and the evolution of Earth's near-surface environments. *Econ. Geol.* **100**, 1489–1509 (2005).

50. N. Lotem, B. Rasmussen, J.-W. Zi, S. S. Zeichner, T. M. Present, Y. M. Bar-On, W. W. Fischer, Reconciling Archean organic-rich mudrocks with low primary productivity before the Great Oxygenation Event. *Proc. Natl. Acad. Sci. U.S.A.* **122**, e2417673121 (2025).

51. P. Glynn, Solid-solution solubilities and thermodynamics: Sulfates, carbonates and halides. *Rev. Mineral. Geochem.* **40**, 481–511 (2000).

52. P. Bénézeth, J. L. Dandurand, J. C. Harrichoury, Solubility product of siderite ($FeCO_3$) as a function of temperature (25-250°C). *Chem. Geol.* **265**, 3–12 (2009).

53. P. Bénézeth, G. D. Saldi, J.-L. Dandurand, J. Schott, Experimental determination of the solubility product of magnesite at 50 to 200°C. *Chem. Geol.* **286**, 21–31 (2011).

54. E. A. Guggenheim, Theoretical basis of Raoult's law. *Trans. Faraday Soc.* **33**, 151–159 (1937).

55. A. N. Awolayo, B. M. Tutolo, PyGeochemCalc: A Python package for geochemical thermodynamic calculations from ambient to deep Earth conditions. *Chem. Geol.* **606**, 120984 (2022).

56. E. L. Shock, E. H. Oelkers, J. W. Johnson, D. A. Sverjensky, H. C. Helgeson, Calculation of the thermodynamic properties of aqueous species at high pressures and temperatures. Effective electrostatic radii, dissociation constants and standard partial molal properties to 1000 °C and 5 kbar. *Faraday Trans.* **88**, 803–826 (1992).

57. C. M. Bethke, B. Farrell, S. Yeakel, *The Geochemist's Workbench Release 12: GWB Essentials Guide*, 186 (Aqueous Solutions LLC, 2022).
